# Microbiota and Colorectal Cancer: From Gut to Bedside

**DOI:** 10.3389/fphar.2021.760280

**Published:** 2021-09-30

**Authors:** Miguel Silva, Valentina Brunner, Markus Tschurtschenthaler

**Affiliations:** ^1^ Institute of Molecular Oncology and Functional Genomics, Center for Translational Cancer Research (TranslaTUM), Klinikum Rechts der Isar, Technical University of Munich, Munich, Germany; ^2^ Graduate Program in Areas of Basic and Applied Biology (GABBA)/ICBAS - Institute for the Biomedical Sciences Abel Salazar, Porto University, Porto, Portugal; ^3^ Institute for Experimental Cancer Therapy, Center for Translational Cancer Research (TranslaTUM), Klinikum Rechts der Isar, Technical University of Munich, Munich, Germany; ^4^ Department of Internal Medicine II, Klinikum Rechts der Isar, Technical University of Munich, Munich, Germany

**Keywords:** colorectal cancer, microbiota, dysbiosis, host-microbiota interactions, therapy

## Abstract

Colorectal cancer (CRC) is a complex condition with heterogeneous aetiology, caused by a combination of various environmental, genetic, and epigenetic factors. The presence of a homeostatic gut microbiota is critical to maintaining host homeostasis and determines the delicate boundary between health and disease. The gut microbiota has been identified as a key environmental player in the pathogenesis of CRC. Perturbations of the gut microbiota structure (loss of equilibrium and homeostasis) are associated with several intestinal diseases including cancer. Such dysbiosis encompasses the loss of beneficial microorganisms, outgrowth of pathogens and pathobionts and a general loss of local microbiota diversity and richness. Notably, several mechanisms have recently been identified how bacteria induce cellular transformation and promote tumour progression. In particular, the formation of biofilms, the production of toxic metabolites or the secretion of genotoxins that lead to DNA damage in intestinal epithelial cells are newly discovered processes by which the microbiota can initiate tumour formation. The gut microbiota has also been implicated in the metabolism of therapeutic drugs (conventional chemotherapy) as well as in the modulation of radiotherapy responses and targeted immunotherapy. These new findings suggest that the efficacy of a given therapy depends on the composition of the host’s gut microbiota and may therefore vary from patient to patient. In this review we discuss the role of host-microbiota interactions in cancer with a focus on CRC pathogenesis. Additionally, we show how gut bacteria can be exploited in current therapies and how mechanisms directed by microbiota, such as immune cell boost, probiotics and oncolytic bacteria, can be applied in the development of novel therapies.

## Introduction

### Gut Microbiota: The Neighbours We Need

In an effort to better characterize bacteria in humans, the Human Microbiome Project (HMP) was established in 2008 whose main mission is the generation of a database to allow extensive analysis of the human microbiome and assess its role in health and disease (Human Microbiome Project, 2012). The natural human microbiome consists of a large collection of several microorganisms from viruses to prokaryotes (archaea and bacteria) as well as eukaryotes. In total, a human adult harbours the same number of bacterial cells as its own human cells (Sender et al., 2016). However, the number of genes encoded by these bacteria sums up to a striking number of about 2 million genes (Simon and Gorbach, 1984). The widespread colonization of bacteria in the intestine starts right after birth and results in a gradient along the gastrointestinal tract with increasingly more colony forming units (CFU) from the proximal small intestine to the colon (Sender et al., 2016). Most microbial taxa identified from human stool samples are members of the phyla of *Firmicutes*, *Bacteroidetes*, *Proteobacteria*, *Actinobacteria* and *Verrucomicrobiota*. Over 90% of them belong to *Firmicutes* or *Bacteroidetes* phyla mostly represented by the genera *Veillonella* or *Bacteroides*, respectively. Although the fraction of these two phyla can differ between individuals, the quantity of genes encoding functionality or metabolic functions remains stable (Human Microbiome Project, 2012). Large scale comparisons including samples from different subjects sampled from different places in the intestine show not only differences between individuals, but also between anatomical sites within the intestine (Eckburg et al., 2005; Human Microbiome Project, 2012).

**FIGURE 1:**
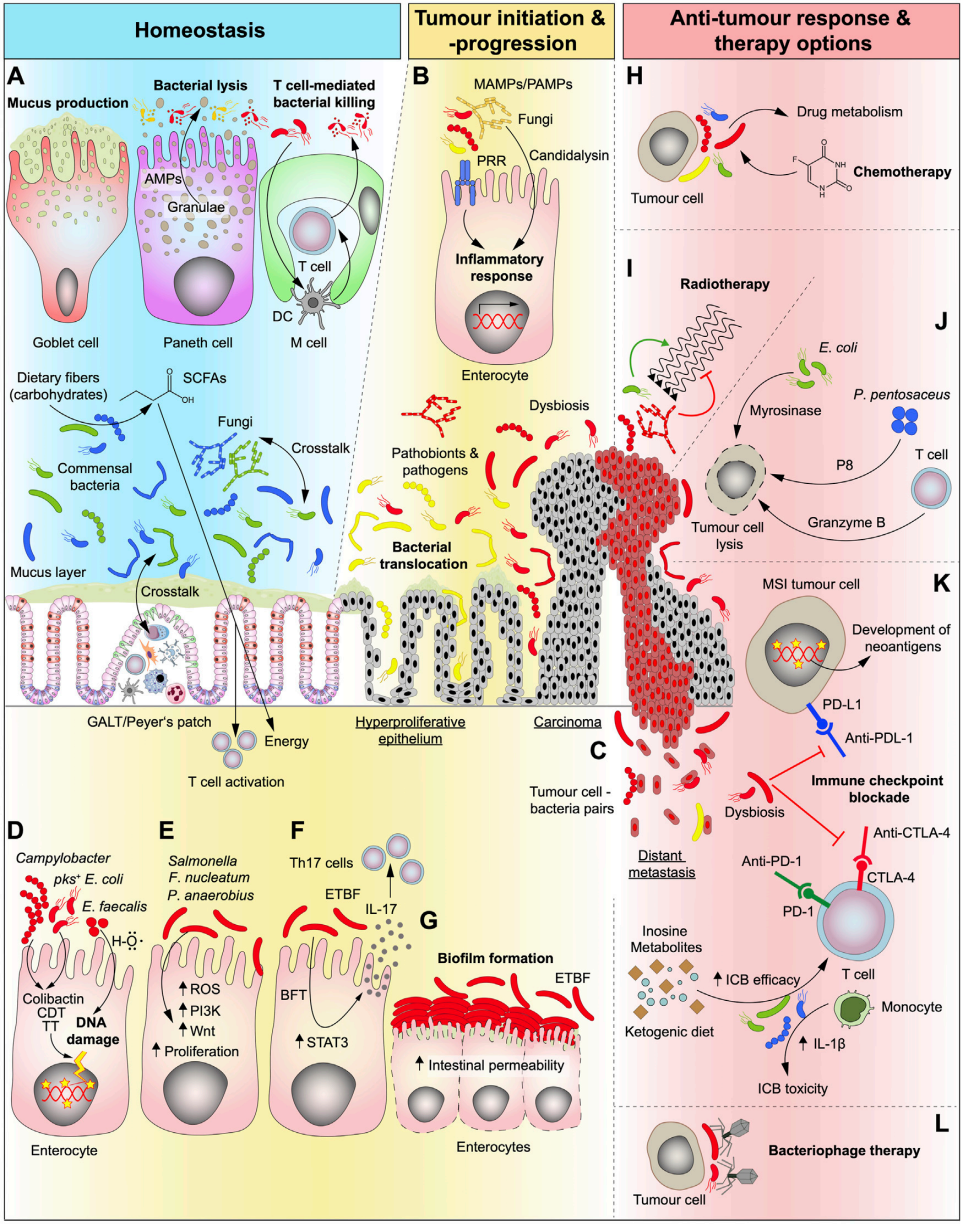
Role of the gut microbiota under homeostatic conditions and in different stages of CRC development as well as its relevance and potential in conventional and future therapy options. **(A)** In the healthy gut, bacteria are essential for the digestive process by breaking down complex foods into metabolites that can be absorbed by the human organism. Carbohydrates are converted by bacteria into short-chain fatty acids (SCFAs) which are an energy source that can be absorbed by the human gut. The intestine is “equipped” with a plethora of mechanisms that synergistically act to help maintain a healthy microbiome composition and to separate fungi and bacteria from the host cells. Goblet cells produce mucus that serve as physical barrier keeping bacteria separated from epithelial cells and Paneth cells kill opportunistic pathogens by the secretion of antimicrobial peptides (AMPs). Microfold cells (M cells) are found in the gut-associated lymphoid tissue (GALT) of the Peyer’s patches and can translocate B and T cells to the intestinal lumen in order to kill bacteria. They can also present bacterial antigens to dendritic cells (DC) and elicit an IgA-specific immune response. **(B)** In the transformation of normal to malignant tissue, microbiota is dysregulated (dysbiosis) and activate several cell-intrinsic mechanisms that fuel tumour progression. Microbe- and pathogen-associated-molecular-patterns (MAMPs/PAMPs) activate the innate immune system through pattern-recognition-receptors (PPR) resulting in an inflammatory response in epithelial cells. **(C)** As disease progresses, bacteria were shown to seed together with tumour cells to other organs. **(D)** Genotoxic bacteria produce toxins such as Colibactin, cytolethal distending toxin (CDT) and typhoid toxin (TT) or hydroxyl radicals (H–O) and induce DNA damage and may serve as the initiating event of a malignant transformation. *pks*, polyketide-nonribosomal peptide synthase operon. **(E,F)** Dysregulated microbiota can interact directly with epithelial and immune cells and activate CRC-related pathways, such as phosphatidylinositol-3-kinase (PI3K) and Wnt, involved in proliferation and cell survival **(E)** thereby kickstarting cellular transformation. Additionally, some genotoxins can also upregulate signal transducer and activator of transcription 3 (STAT3) pathway which leads to proliferation and T cell activation and can thereby elicit a Th17 immune response **(F)**. ROS, reactive oxygen species; ETBF, enterotoxigenic *Bacteroides fragilis*; BFT*, Bacteroides fragilis* toxin. **(G)** Accumulation of certain bacteria strains (biofilm formation) can be detected at sites of both normal and tumour tissue which can disrupt the epithelial barrier. **(H,I)** Conventional cancer therapy (radiotherapy and chemotherapy) efficiencies can be modulated by the commensal intestinal bacteria and fungi and the resulting side effects can be, attenuated. Bacterial metabolism of chemotherapeutic drugs impacts both the efficiency and the development of side effects from therapy **(H)**. While bacteria can enhance the effects of radiotherapy, fungi hamper its efficiency **(I)**. **(J)** Gut microbiota can be modulated to halt tumour progression and kill malignant cells, directly, by loading bacteria with cytotoxic cargos, such as P8 or myrosinase, or, indirectly, by stimulating immune cells. **(K)** Immune checkpoint blockade (ICB) drugs are greatly influenced by bacteria (and fungi). The presence of a given bacterial strain can increase therapy efficiency by direct stimulation of immune cells or by production of intermediate metabolites. Dysbiosis can also impair therapy efficiency and at the same time lead to therapy-related toxicity events resulting from the treatment. The presence of a hypermutated phenotype as seen in microsatellite instable (MSI) tumours leads to the generation of neoantigens at the tumour cell surface that are associated with better ICB therapy outcomes, which can additionally be influenced by the gut microbiota. The presence of a dysbiotic microbiota can inhibit the effects of ICB. **(L)** Another way to stop tumour progression could be by targeting harmful tumour-promoting bacterial strains with highly specific (designed) bacteriophages.

Bacteria have been shown to play a crucial role in the digestive process taking place in the gut and thus in many metabolic processes responsible for energy production in the human organism (Figure 1A). For instance, carbohydrates are fermented and synthesized into short-chain fatty acids (SCFAs) by organisms belonging to the groups of *Bacteroides*, *Bifidobacterium*, *Enterobacteria*, *Fecalibacteria* and *Roseburia* which provide a source of energy to the host (Macfarlane and Macfarlane, 2003). SCFAs can additionally regulate the activity of immune cells by promoting expansion of regulatory T cells (Tregs) and by improving the activity of effector T cells (Luu et al., 2021; Smith et al., 2013) (Figure 1A). Gut bacteria are also essential for the transformation of natural compounds present in the human diet. Lignans, for instance, are present in foods such as flaxseeds, vegetables and fruits and its bioconversion by bacteria renders them possible to be digested and absorbed by the human organism, where they were shown to have a protective effect against cancer and other diseases (Landete, 2012; Fuentealba et al., 2014). The conversion of lignans into secoisolariciresinol diglucoside (SDG) and the subsequent production of enterodiol (ED) and enterolactone (EL), which are responsible for the beneficial effects of lignans, involves a very complex series of steps. Eleven bacterial strains were identified to be responsible for these series of processes including several species from the genus *Clostridium* and *Bacteroides* and individual species such as *Eubacterium limosum*, *Peptostreptococcus productus* and *Eggerthella lenta* (Clavel et al., 2006). Isoflavones can be obtained from soy-based foods and are then metabolized in the gut by certain bacterial strains such as *Adlercreutzia equolifaciens*, *Enterorhabdus mucosicola* and *Slackia isoflavoniconvertens* (Vázquez et al., 2020). Also, the resulting metabolite from this interaction (O-desmethylangolensin) has been suggested to bear a protective role in a variety of diseases including cancer, cardiovascular disease and osteoporosis (Atkinson et al., 2005).

Therefore, it is not surprising that the gut microbiota is highly dependent and influenced by dietary preferences (David et al., 2014). In an elegant study from David et al. (2014) the authors were able to address the effects of two very distinct diets: a “plant-based” diet rich in grains, legumes, fruits and vegetables, and an “animal-based” diet, mostly comprising meat, eggs and cheese (David et al., 2014). By administering these diets for 5 consecutive days on human test subjects, the authors reported shifts in microbiota composition that were specifically associated with one diet or the other. For instance, the animal-based diet increased the abundance of bile-tolerant microorganisms (*Alistipes, Bilophila* and *Bacteroides*) and decreased the levels of *Firmicutes* that metabolize dietary plant polysaccharides (*Roseburia*, *Eubacterium rectale* and *Ruminococcus bromii*). Moreover, the former diet led to increased production of bile acids such as deoxycholate (DCA) which were previously described to promote cancer through mitochondrial oxidative stress (Payne et al., 2007; Yoshimoto et al., 2013). More recently, the association between diet and gut microbiome and its effect on disease predisposition was reported in a multi-generational Asian-American immigrant cohort (Vangay et al., 2018). Migrants from rural areas of Asia to the United States were followed up over generations, together with their dietary and microbial changes. Comparison of the microbiome before and after migration showed changes associated with lower phylogenetic diversity and function, reduction of *Prevotella* strains and particularly of fiber-degrading enzymes. Interestingly, the number of obesity cases increased after migration (Vangay et al., 2018).

Considering the fact that the gut is the organ with the greatest immunological portfolio, the colonization of over 400 different bacterial species along the intestine is quite striking, which suggests an important commensal relationship between the two entities (Simon and Gorbach, 1984). Several studies have shown that the gut microbiota is able to regulate the differentiation and expansion of different types of T cells (IvanovAtarashi et al., 2009; Atarashi et al., 2011; Round and Mazmanian, 2010). Nonetheless, not all bacteria bear a protective or supportive role to the host, which is a reason why the human body has developed several mechanisms to adapt and “learn” to co-exist with bacteria. One of the most important processes in this equilibrium is the formation of a barrier that serves as a physical and chemical wall that separates bacteria from intestinal epithelial cells (Clevers and Bevins, 2013). Paneth and goblet cells are the most important intervening cell types in the mucus barrier formation, by producing granule filled with MUC2, glycosylated mucin glycoproteins, lipids as well as antimicrobial enzymes, and immunoglobulins (Figure 1A) (Johansson et al., 2011). The mucus functions as a physical wall that separates intestinal epithelial cells (IECs) from the luminal content of the intestine. Besides protecting intestinal cells against chemical and mechanical insults, it also helps in the removal of debris and bacteria, mediating at the same time the diffusion of small molecules such as ions, water and nutrients to the enterocytes (Paone and Cani, 2020). The gradient of bacteria in the intestine reflects also a gradient of thickness of the barrier along the intestine, increasing from ∼120 µm in the jejunum to the ∼830 µm in the colon. While the small intestine only harbours a rather loose layer, the colonic mucus barrier comprises a firmly attached and impermeable inner stratified layer. The more dynamic outer layer however, provides loose glycans to nourish microbial habitants (Atuma et al., 2001; Johansson et al., 2011).

For homeostasis to occur in the gut, constant flow of information needs to be assured between bacteria, IECs and immune cells. A rupture in this delicate balance might lead to bacteria invading the mucus layer and reaching direct contact with epithelial cells. Once the mucus layer is conquered by potentially pathogenic bacteria, pattern recognition receptors (PRRs) such as toll-like receptor (TLR) on IECs are activated by microbe-associated molecular patterns (MAMPs) which in turn activate a plethora of innate immune reactions resulting in inflammatory and immunomodulatory responses (Figure 1B). At the same time, Paneth cells, which are located in the crypt base of the small intestine, constantly release antimicrobial peptides (AMPs) (Figure 1A). These molecules such as defensins, lysozymes and C-type lectins feature an unspecific but effective approach in protecting stem cells in their niche (Sansonetti, 2004). In humans, the most abundant AMPs belong to the group of defensins such as α-defensin, which permeabilize the plasma membrane of Gram-negative and Gram-positive bacteria (Ganz, 2003). Indeed, impaired Paneth cell function was shown to not only result in dysbalanced microbiota but also in chronic inflammation, suggesting that Crohn’s disease (CD) is a specific disorder of this cell type (Adolph et al., 2013; Tschurtschenthaler et al., 2014). Additional maintenance of homeostasis is given by the gut-associated lymphoid tissue (GALT) (Figure 1A). GALT produce a special type of immunoglobulins (IgA) that are released into the intestinal lumen and neutralizes potential pathogens independent of the complement system (Gutzeit et al., 2014). This process is mainly mediated by M cells which are commonly found overlying organized mucosal lymphoid tissues such as Peyer’s patches. M cells, or microfold cells, like the other cells in the intestinal epithelium, derive from stem cells located at the bottom of the crypt, however, display different morphological features. For instance, M cells are devoid of apical microvilli, and present a basolateral pocket harbouring usually a B or T cell that can be translocated to the lumen and direct their action towards pathobionts such as viruses and bacteria (Figure 1A). These features are crucial for the mode of action of M cells regarding immunosurveillance (Dillon and Lo, 2019). Moreover, M cells specialize in the uptake and delivery of bacterial or dietary antigens from the intestinal lumen to antigen presenting cells (APCs), such as dendritic cells (DCs), located in the lamina propria. This in turn leads to activation of B and T cells and induces, the already mentioned, antigen-specific IgA production at the mucosa level (Figure 1A) (Okumura and Takeda, 2017).

## Microbial Perturbations in Colorectal Cancer Development

Given the high complexity of biological processes involved in maintaining a healthy environment between bacteria and human tissues, it is not surprising to find that perturbations in microbiota composition (dysbiosis) are seen in several pathological conditions including cancer (Biragyn and Ferrucci, 2018). Dysbiosis relates to any change in the composition of resident gut bacteria and fungi in comparison to the community found in healthy individuals. Moreover, it encompasses the loss of beneficial microorganisms, expansion of harmful microbes (pathobionts and pathogens), a general loss of local microbiota diversity and bacterial translocation across the epithelial barrier (Petersen and Round, 2014; Genua et al., 2021) (Figure 1B).

The association of the microbiota with cancer has recently been highlighted in a study that showed that different cancer types present distinct microbial signatures. Strikingly, bacteria were also found to be physically present inside the tumour cells as well as in immune cells of cancer patients (Nejman et al., 2020). This suggests that certain bacteria have a predilection for certain tissue types, likely because these tissues constitute the right environment therefore providing a growth advantage to a given bacterial community. However, the causal nature of these findings remains elusive, since only now we started to understand how bacteria interact with tumour and immune cells to boost tumour growth.

Although no disease-specific microbiota signature has been identified, patients with CRC have shown reduced bacterial diversity and richness compared to healthy individuals. *Firmicutes* and *Bacteroidetes* are enriched in CRC (Nejman et al., 2020; Yachida et al., 2019). Moreover, changes in the diversity of bacterial communities in the gut is a common event in colorectal tumours. Specific bacteria, such as *Fusobacterium nucleatum* and several microbial metabolites have been associated with the onset and progression of this disease (Yachida et al., 2019; Castellarin et al., 2012; Kostic et al., 2012; Wu et al., 2021; Feng et al., 2015). Using faecal samples from a large cohort of CRC patients in different stages of disease progression Yachida et al. (2019) conducted the largest metagenomics (*n* = 616) and metabolomics (*n* = 406) analysis on human CRC to date (Yachida et al., 2019). The authors were able to pinpoint specific shifts in bacterial composition, bacterial gene abundance and associated metabolites, and map them to different stages of tumour progression. Some elements of the phyla *Firmicutes*, *Fusobacteria* and *Bacteroidetes* are predominantly elevated in carcinoma patients compared to adenomas and healthy controls, whereas species such as *Atopobium parvulum*, *Actinomyces odontolyticus*, *Desulfovibrio longreachensis* and *Phascolarctobacterium succinatutens* are mostly elevated in early stages of disease (Yachida et al., 2019). In another study with a similar approach, the authors performed metabolomic shotgun sequencing on faecal samples from 55 healthy controls, 42 advanced adenoma and 41 carcinoma patients (Feng et al., 2015). Interestingly, there was a significant increase of *Bacteroides* such as *B. dorei* and *B. massiliensis* from healthy to advanced adenoma, and an increase of *B. massiliensis*, *B. ovatus*, *B. vulgatus* and *E. coli* from advanced adenoma to carcinoma. Concomitantly, the lactic acid-producing bacteria *Bifidobacterium animalis*, *Streptococcus mutans* and *Streptococcus thermophilus* were enriched in control faecal samples only. Lactate production was shown to accelerate colon epithelial cell turnover and to maintain gut homeostasis. The decrease in lactic acid-producing commensals caused by dysbiosis, could lead to progression of early to advanced disease stages (Feng et al., 2015). More recently, bacterial communities were also shown to vary their composition during CRC progression (Wu et al., 2021). By comparing the microbiome in stool samples from 306 adenoma patients, 217 CRC subjects and 252 healthy controls, the authors identified 43 bacteria species with distinguishable differential abundances between controls and patients with adenoma. Similarly, 114 differentially abundant bacteria were detected between adenoma and cancer (Wu et al., 2021). Of note, *Fusobacterium nucleatum* was enriched in cancer samples compared to adenoma further confirming previous findings (Yachida et al., 2019). In fact, several studies have suggested a pro-tumorigenic effect of *F. nucleatum* in CRC both *in vitro* and *in vivo* (Yang et al., 2017; Kostic et al., 2013). Using a cohort of human primary CRC and paired hepatic and lymph node metastasis, Bullman et al. (2017) showed the presence of *F. nucleatum* not to be restricted to the primary site of disease (primary tumour) but also at distant sites (metastases). Notably, whole genome sequencing (WGS) analysis and culture of bacteria isolates from tumour-metastasis pairs showed almost complete sequence identity between both sites, strongly indicating that bacteria could indeed migrate with tumour cells to metastatic sites or even facilitate the metastatic process (Bullman et al., 2017) (Figure 1C). Interestingly, only *F. nucleatum-*positive tumours derived from CRC patients successfully engrafted in mice (PDX model). Treatment of the latter with metronidazole (a drug known to kill *Fusobacteria*) resulted in decreased tumour growth compared to controls while no effect of the drug was seen in *Fusobacterium*-negative xenografts (Bullman et al., 2017). Although proof of a direct stage-specific functional effect of the microbiota on human tumour lesions remains to be uncovered, it is reasonable to assume that CRC progression can be influenced by the microbiota and its metabolites, thereby adding an extra layer of complexity to the classical adenoma-carcinoma progression model (Fearon and Vogelstein, 1990).

The combined data from the aforementioned studies together with the decrease in the costs of sequencing technologies over time, suggests the possibility of using the gut bacteria for diagnostic assessment of disease. In fact, the idea of using bacteria as a non-invasive way to predict disease has been explored in several recent studies and the results seem indeed promising (Liang et al., 2020; Poore et al., 2020; Wu et al., 2021). Remarkably, by combining the data of the differentially enriched bacteria in different stages of disease together with patient data (such as age, gender and BMI) the authors could successfully distinguish adenoma from healthy control subjects (AUC 0.8) and adenoma from cancer samples (AUC 0.89) (Wu et al., 2021). Concomitantly, metagenomic analysis of the faecal microbiome of a large cohort of normal controls, adenoma and carcinoma samples allowed the identification of a bacterial gene marker (*m3*) from a *Lachnoclostridium sp*. for the diagnosis of colorectal adenoma (Liang et al., 2020). By combining this marker with the presence of *Fusobacterium nucleatum*, *Clostridium hathewayi*, *Bacteroides clarus* and faecal immunochemical test (FIT) the authors reported a sensitivity of 94% for diagnosing CRC (Liang et al., 2020). Notably, by looking at the bacterial sequences of available TCGA data from many distinct cancer types and controls, Poole et al. (2020) developed a machine learning approach which allowed the discrimination between cancer types (*n* = 32), and from tumour and normal samples in 15 different cancer types. The strength of the pipeline was validated by applying the same procedure on WGS sequencing data of circulating tumour DNA (ctDNA). Once more, the approach proved to be very efficient in differentiating distinct tumour types even for early-stage disease and for cancers that do not show any genomic alterations, thus overcoming one of the main limitations of current existing ctDNA assays (Poore et al., 2020).

We only recently started to unveil the great potential of using bacteria as a diagnostic tool in CRC and other cancer types. Hence, several technical limitations should be taken into account in the process, including the possibility of sample contamination during collection, sample processing, and overall sequencing costs. It is therefore imperative that proper procedural controls are included in the study design in order to minimize the contributions of contaminants to microbial signatures and prevent interpretation bias of the results (Salter et al., 2014; Eisenhofer et al., 2019).

Besides bacteria, the intestinal tract is also colonized by a variety of fungal species that make up for approximately 0.001–0.1% of the total gut microbiota. The most common phyla that constitute the gut mycobiome belong to *Basidiomycota, Glomeromycota* and *Ascomycota,* and like bacteria, fungal dysbiosis has also been associated with disease (Liguori et al., 2016; Luan et al., 2015). Notably, several studies have reported changes in gut fungi communities in CRC and inflammatory bowel disease (IBD) patients in comparison to healthy individuals (Luan et al., 2015; Gao et al., 2017; Coker et al., 2019). Nonetheless, no disease-specific mycobiome signature has so far been identified. One of the ways by which fungi can contribute to inflammation and ultimately CRC, comes from the observations that *Candida albicans* and *Candida tropicalis* are increased in inflammation and cancer settings. These two fungal species produce a cytosolic peptide called candidalysin, known to promote the disruption of the epithelial barrier function (Qin et al., 2021) (Figure 1B). It was suggested that toxic metabolites and other substances produced by fungi could lead to disruption of cell-intrinsic pathways as well as impair immune signalling in order to drive disease. Additionally, the crosstalk between fungi and bacteria adds another perspective to the current state of knowledge in the field, as it has been shown that these two entities can influence each other on different levels (Vallianou et al., 2021) (Figure 1A). Further studies are needed, however, to complement the existing information regarding the role of the mycobiome in disease aetiology. The current knowledge on the association of fungi in different cancer types has been recently reviewed elsewhere (Vallianou et al., 2021).

As previously stated, viruses are also a component of the gut microbiota and their role in disease is well elucidated. Viruses are able to infect a plethora of human tissues including the upper respiratory tract and lungs, the colon, liver and blood cells. Upon infection, they are able to induce signalling in host cells and control processes such as growth and survival which are usually altered in cancer (Mesri et al., 2014). Another way through which viruses can induce cell transformation is by inducing the DNA damage response machinery in infected cells causing genomic instability which increases mutation rate (McFadden and Luftig, 2013). Finally, viruses can induce chronic inflammatory reactions which increase the levels of cellular stress and reactive oxygen species (ROS) ultimately leading to the acquisition of mutations (Arzumanyan et al., 2013). It is estimated that almost 10% of cancer cases are caused by viruses, and to date seven viruses have been identified with a strong link to several cancer types (Plummer et al., 2016). Of note, Epstein-Barr virus (EBV) increases the risk of Burkitt lymphoma and other lymphoma types as well as gastric cancer (Young et al., 2016), while hepatitis B (HBV) and C virus (HCV) infections can lead to liver cancer (El-Serag, 2012). Some viruses such as Merkel cell polyomavirus, human herpes virus 8 (HHV-8), human papillomavirus (HPV) and human T-cell leukemia virus type (HTLV-1) can be sexually transmitted and cause several types of cancer including lymphomas and leukaemia, Kaposi sarcoma and carcinomas (Krump and You, 2018).

## Mechanisms of Colorectal Cancer-Promoting Bacteria

Although bacteria are necessary for many biological processes in the organism, they also contribute to different pathological states when dysregulated. New mechanisms have been discovered by which bacteria and their metabolites or toxins can cause direct DNA damage and induce oncogenic mutations. For instance, *E. faecalis* infection leads to increased production of hydroxyl radicals which are known to cause DNA damage (Wang and Huycke, 2007) (Figure 1D). On the other hand, *P. anaerobius,* which is increased in human colon tumours, increases ROS levels leading to higher cholesterol levels and increased cell proliferation in colon cancer cells (Tsoi et al., 2017) (Figure 1E). Additionally, *P. anaerobius* can also selectively adhere to the CRC mucosa *in vivo* and promote tumour growth in a process mediated by its surface protein, putative cell wall binding repeat 2 (PCWBR2). In this scenario, CRC progression is thought to occur due to the interaction of bacterial PCWBR2 with eukaryotic cells’ α2/β1 integrin, which induces the activation of the PI3K–AKT pathway in cancer cells (Figure 1E). This in turn leads to increased cell proliferation and NF-κB-driven inflammation. Surprisingly, the resulting effects from this interaction were greatly attenuated or even absent in normal colonic cells (Long et al., 2019). *Salmonella* infection in humans can become chronic if not treated properly and was shown to cause low-grade but persistent inflammation. Infection with an AvrA-expressing *Salmonella* strain of an inflammation-driven CRC mouse model led to increased activation of STAT3 and Wnt signalling pathway thus leading to an increased proliferation and tumourigenesis in these animals (Lu et al., 2016) (Figure 1E).

Mounting evidence points to the fact that bacteria can sense stage specific features of cancer cells and use those as signalling cues for their own advantage. In line with this, one of the possible mechanisms by which *Fusobacterium nucleatum* may contribute to the progression of CRC has been reported by Rubinstein et al. (2019). *F. nucleatum* interacts with cancer cells through its adhesin molecule FadA which in turn binds eukaryotic Annexin A1 in a process mediated by E-cadherin. Furthermore, the authors showed that FadA, Annexin A1 and E-cadherin form a protein complex with β-catenin thereby modulating its expression (Rubinstein et al., 2019). β-catenin is a central effector of Wnt pathway which is dysregulated in the majority of CRC (Dekker et al., 2019) (Figure 1E).

In addition to the aforementioned mechanisms, bacteria were shown to produce genotoxins such as colibactin, cytolethal distending toxin (CDT) and typhoid toxin (TT) that are able to induce DNA damage *in vitro* and fuel CRC *in vivo* (Cuevas-Ramos et al., 2010; Arthur et al., 2014; Cougnoux et al., 2014; Martin et al., 2019) (Figure 1D). For instance, CDT is composed of three subunits CdtA, CdtB and CdtC, and has the ability to induce host DNA damage due to its DNase I-like property (He et al., 2019). The association between the CDT-producing bacteria *Campylobacter* spp. with cancer and inflammation has been reported (Gradel et al., 2009; Allali et al., 2015). More recently *Campylobacter* spp. was also shown to directly promote tumorigenesis in a CRC mouse model, in a process dependent on the production of CDT (He et al., 2019). On the other hand, colibactin is produced by *E. coli* from a 50-kb hybrid polyketide-nonribosomal peptide synthase operon (*pks*) encoded in its genome. It has been shown that colibactin causes DNA double-strand breaks and activation of the DNA damage checkpoint pathway that can lead to cell death both *in vitro* (Nougayrède et al., 2006) and *in vivo* (Cuevas-Ramos et al., 2010) (Figure 1D). Furthermore, an association between this toxin and cancer (Dejea et al., 2018) and IBD (Arthur et al., 2012) has been reported. In an effort to address the effects of *pks* ^*+*^ *E. coli* in the transformation of epithelial cells of the human colon, Pleguezuelos-Manzano and colleagues (Pleguezuelos-Manzano et al., 2020) microinjected this bacteria strain into the lumen of human clonal intestinal organoids. WGS analysis of the latter enabled the identification of a very distinct mutational signature in the infected organoids compared to controls. Surprisingly, this signature was also shown to be present in human cancers (Pleguezuelos-Manzano et al., 2020). These results were recently corroborated in murine organoids (Iftekhar et al., 2021). As such, infection of mouse normal colon organoids with *pks* ^*+*^ *E. coli* leads to increased DNA damage, megalocytosis, formation of multinucleated cells as well as mutations in common cancer-associated genes. Surprisingly, infected organoids lost their dependence on medium Wnt agonists by upregulating Wnt/β-catenin signalling while downregulating differentiation genes such as carbonic anhydrase 4 (*Car4*) and aquaporin 8 (*Aqp8*) (Iftekhar et al., 2021). In line with this, another study showed that targeting the metabolism of malignancy-promoting colibactin-producing *E. coli* strains in the gut microbiota in an AOM/DSS cancer mouse model, reduces the risk of CRC development (Zhu et al., 2019). *Bacteroides fragilis* toxin (BFT) produced by enterotoxigenic *Bacteroides fragilis* (ETBF) is also associated with colitis and CRC (Boleij et al., 2015). In fact, colonizing multiple intestinal neoplasia (*Min* ^*ApcΔ716/+*^) mice heterozygous for the *Apc* gene, with ETBF, leads to the formation of tumours in the distal colon in a process mediated by IL-17 (Figure 1F) (Wu et al., 2009). On a molecular level, bacterial infection led to STAT3 activation in the colon of these animals which in turn leads to a Th17 response characterized by IL-17-secreting CD3^+^CD4^+^ and CD3^+^CD4^–^ cells (Figure 1F). The Th17 response is associated with tumour progression through its role in immunosuppression and angiogenesis (Asadzadeh et al., 2017; Salazar et al., 2020). Conversely, blocking IL-17 production in the intestine attenuates tumorigenesis in this mouse model (Wu et al., 2009).

More recently, introduction of a *BRAF* ^*V600E*^ mutation into the pre-existing *Min* ^*ApcΔ716/+*^ ETBF mouse model (Wu et al., 2009) caused a shift in the spectrum of colon tumours towards a more proximal location within the colon (DeStefano Shields et al., 2021). In addition, these tumours displayed a serrated-like histopathology and were characterized by a strong IFNλ-driven immune signature. Notably, this signature was associated with a recruitment of PD-L1 expressing myeloid-derived suppressor cells (MDSC) and CD8^+^ tumour infiltrating T cells. In line with this, treatment of tumour-bearing mice with PD-L1 blockade therapy led to reduced colon tumour numbers in this model (DeStefano Shields et al., 2021). Additionally, loss of membrane-associated E-cadherin*, in vitro* was reported after treatment of a colon cancer cell line with BFT (Wu et al., 2003). However, the exact biological mechanisms supporting the association CRC and ETBF in humans requires further studies.

Another mechanism by which the gut microbiota can promote CRC is the formation of biofilms, which are aggregates of bacteria encased in a polymeric matrix mostly present on right-sided colon tumours (Dejea et al., 2014). Interestingly, biofilms increase the permeability of the intestinal barrier allowing bacterial invasion and at the same time increasing proliferation of the tissue (Dejea et al., 2014). The association between biofilms formation and sporadic human CRC has been reported (Dejea et al., 2014; Johnson et al., 2015). In the first effort to associate bacterial biofilms with CRC, Dejea and colleagues (2014) showed that the presence of invasive polymicrobial bacterial biofilms is associated mostly with right sided tumours in a cohort of CRC patients. Strikingly, patients with biofilm-positive lesions also showed the presence of biofilms in the normal intestinal mucosa far from the tumour (Dejea et al., 2014). In addition, intestinal barrier integrity was compromised in biofilm-positive samples. Although global expression levels of E-cadherin were not altered between biofilm-positive and biofilm-negative samples, there was a shift of the marker expression to the basal pole of the epithelial cells in the former samples, which was consistent with the observation of increased permeability of the epithelial barrier (Dejea et al., 2014). The same group has later suggested that one way by which bacterial biofilms might affect tumour formation and growth is by the production of the polyamine metabolite N1, N12- diacetylspermine which was found to be upregulated in biofilm-positive samples (tumour and healthy tissue) compared to biofilm-negative samples (Johnson et al., 2015). More recently, the association between biofilms and hereditary CRC was also reported (Dejea et al., 2018). In a subset of familial adenomatous polyposis (FAP) patients, biofilms were mainly composed of *Proteobacteria* and *Bacteroides*, with an overrepresentation of *E. coli* and *B. fragilis* species (Dejea et al., 2018). The presence of colibactin and BFT produced by the above-mentioned bacterial strains, respectively, was significantly associated with the mucosa of FAP patients (Figure 1G). In order to validate these findings, the authors colonized the colon of two distinct CRC mouse models with *E. coli* and *B. fragilis*, which led to faster tumour growth and increased mortality of the animals (Dejea et al., 2018).

Malignant transformation of eukaryotic cells is a very complex biological process that is influenced mainly by genetic and epigenetic cues that come both from within the cell but also from cell-extrinsic mediators such as stromal and immune cells (Hanahan and Weinberg, 2011). The study of the microbiome in the cancer setting suggests that a new variable must be included in this relationship given the mutagenic abilities of some bacteria that inhabit the human gut. Only recently we started to unravel how bacteria can indeed directly influence cell pathways and how this leads to a disease state. Given the tremendous abundance and diversity of bacteria in the human organism it is likely that many other mechanisms will be identified in the years to come.

## The Role of the Microbiota in Drug Metabolism

With a ratio of approximately 1:1 of bacteria and cells in the human body, these microorganisms additionally encode for 150-fold more genes than the human genome (Sender et al., 2016). The identification of microbiota-specific metabolic signatures deepens the understanding of the relationship between bacteria and human cells and can help predict the response to a given drug or chemical compound. By using a library of 833 metabolites, Han S. and colleagues (Han et al., 2021) identified the metabolic profiles of 178 gut bacteria through a combination of mass spectrometry and a machine learning pipeline. Studies like the aforementioned are able to comprehensively map genes to metabolic features of bacteria and ultimately link this data to phenotypic bacterial variation which can be greatly explored in the context of therapy. Since all orally administered compounds are primarily absorbed in the gut, together with the liver these are the places where most of the metabolic transformation of therapeutic drugs occurs (Foti et al., 2015). It is important, however, to address the bidirectional relation between drug compounds and microbiota since drugs have a stark effect on microbiota composition and can lead to dysbiosis. On the other hand, it has also been recognized that bacteria have the capacity to metabolize drugs (Maier et al., 2018). Examples of drug-induced toxicity on bacteria are given by anti-diabetics such as metformin (Forslund et al., 2015), proton pump inhibitors (PPIs) (Imhann et al., 2016) and nonsteroidal anti-inflammatory drugs (Rogers and Aronoff, 2016). In an effort to broadly and systematically address these effects, Maier and colleagues (2018) treated 40 different bacteria species with 1,197 drugs belonging to different therapeutic classes, excluding antibiotics (Maier et al., 2018). The authors concluded that nearly 30% of the compounds tested inhibited the growth of at least one bacterial species and speculate that antibiotic resistance might also arise due to microbiota changes when exposed to non-antibiotics (Maier et al., 2018). Distinct additional strategies have been employed to dissect the causes and consequences of drug-microbiota interaction such as gain of function and loss of function genetic screens (Zimmermann et al., 2019a; Zimmermann et al., 2019b) as well as probe enzymatic activity assays in order to identify enzymes responsible for specific drug conversions (Jariwala et al., 2020). To study the effect of the microbiota on drug metabolism of the host, García Gonzalez and colleagues (García-González et al., 2017) used the nematode *C. elegans* as a model. By treating *C. elegans* with 11 different therapeutic drugs while feeding them different bacterial diets they were able to unravel distinct host-microbiota responses to therapy. Dietary *E. coli* and *Comamonas* oppositely affected the response to 5-fluoro-20-deoxyuridine (FUDR), and the topoisomerase I (topo-I) inhibitor camptothecin (CPT) which are two commonly used chemotherapeutics in CRC treatment. Thus, more FUDR-treated animals survived when fed with *Comamonas*, while less survival was reported upon treatment with CPT. The opposite trend for both drugs was reported when *C. elegans* was fed with *E. coli.* Interestingly there were no differences in the efficacy of 5-fluorouracil (5-FU) between the 2 diet regimens evaluated (García-González et al., 2017).

The metabolic capacity of the gut microbiota is a topic of great recent interest as it can help explain the differences in therapy outcomes between patients with similar pathologies treated with the same therapeutic regime. Moreover, the identification of toxic by-products of bacterial drug metabolism could help predict possible side effects in patients under treatment. Given the broad range of effects of compound metabolism by the microbiota that include drug activation (Sousa et al., 2014), inactivation (Haiser et al., 2013) or toxicity (Zimmermann et al., 2019a), the precise identification of the bacteria or bacterial signatures that lead to a certain metabolic outcome poses one of the biggest questions of current disease treatments (Figure 1H). For instance, glucuronidation is a phase II transformation that occurs in the liver and inactivates and detoxifies drugs by conjugating them to glucuronic acid (GlcA). These glucuronides are then transported to the intestine where they are excreted from the human body (Li and Jia, 2013). However, once in the intestine, these compounds can also be reactivated by the removal of the GlcA carried out by gut bacterial β-glucuronidases (GUS) enzymes, which leads to local acute toxicity (Jariwala et al., 2020). For instance, irinotecan (CPT-11) is a potent anticancer drug included in different first line therapy regimens to treat several cancer types including CRC (Kelly and Goldberg, 2005). Side effects such as severe diarrhea are common in patients treated with irinotecan. Irinotecan is converted to its active form, human topoisomerase I poison SN-38 in the liver, and later inactivated by DP-glucuronosyl-transferases by adding a GlcA (SN-38-G) conjugate to the original molecule. In the intestine, this inactive conjugate is reactivated by GUS enzymes, which leads to acute toxicity (Wallace et al., 2015). Using a combination of proteomics and bioinformatic analyses on human stool samples, Jariwala et al. identified the GUS enzymes responsible for SN-38 reactivation in the human gut, which is the toxic metabolite of irinotecan (Jariwala et al., 2020). This approach is scalable to other treatment regimens and may therefore be employed to reveal additional biomarkers for prognostic assessment in the era of personalized medicine.

In another effort to characterize the direct metabolic interactions between microbiota and chemical compounds, Javdan and colleagues (2020) developed a tool to identify metabolites generated by microbiome-derived (MDM) enzymes in a set of 23 orally administered drug compounds in human healthy donors. This study encompasses one of the most comprehensive and technically challenging approaches to date, as it involved several different but complementary methodologies including microbial community cultivation, small-molecule structural analysis, quantitative metabolomics, functional genomics and metagenomics, mouse colonization assays, as well a strong bioinformatic component. The authors elegantly showed the feasibility of this system to identify MDM enzymes in a high throughput fashion using drugs from different groups with very distinct modes of action (Javdan et al., 2020). In a similar approach, Zimmermann *et al.* (2019) measured the *in vitro* capacity of 76 naturally occurring bacteria in the human gut to metabolize 271 orally administered drugs belonging to different groups depending on the mode of action (Zimmermann et al., 2019b). Strikingly, up to two thirds of the drugs examined in this study were shown to be metabolized by at least one of the bacteria included in the study (Figure 1H). Moreover, a given bacteria could metabolize up to 95 different drugs. Using a combination of metabolomics, mass spectrometry and DNA sequence analysis, the authors were able to identify specific drug-metabolizing gene products that are responsible for the conversion of drugs into their metabolites (Zimmermann et al., 2019b). Finally, *in silico* tools have also been developed to allow the identification of drugs and respective metabolites by a specific bacteria species (Mallory et al., 2018), and even to predict toxicity events by integrating information regarding bacteria composition, drug activity and diet preferences (Guthrie et al., 2019).

Increasing evidence puts the gut microbiota in the spotlight when discussing drug metabolism in the human organism, as bacteria and their metabolites can impact pharmacokinetics and pharmacodynamics. This becomes particularly important in the context of therapy. The effect of the microbiota in conventional chemotherapy and immune checkpoint blockade therapy (ICB) will be addressed in the next chapter.

## Microbiota as Modulator of Conventional Colorectal Cancer Therapy

Chemotherapeutics have been used for decades and are still a common first-line treatment approach to treat a variety of human tumours, including CRC (Dekker et al., 2019). Nonetheless, these drugs are likely to cause treatment-related morbidities and mortality in a high percentage of patients (Dekker et al., 2019). Given the fact that CRC occurs in physical proximity to the gut bacteria, recent studies are focusing on how intestinal microbiota modulates the efficacy and toxicity of current chemotherapeutic drugs (Brandi et al., 2006; Stringer et al., 2009; Lin et al., 2012; Iida et al., 2013). Fluoropyrimidine-based chemotherapy in combination with oxaliplatin and irinotecan are the standard first line treatment regimens for unresectable advanced stage CRC (Dekker et al., 2019). It was shown that the use of conventional chemotherapeutic drugs such as irinotecan (Lin et al., 2012), 5-FU (Stringer et al., 2009) and cyclophosphamide (Viaud et al., 2013) causes changes in the microbiota diversity of mice in preclinical models and in human patients. However, it is still not clear how this impacts prognosis as some studies show opposing results regarding the effects of microbiota in therapy. For instance, germ-free mice tolerate higher doses of irinotecan and exhibit less gastrointestinal damage as a side effect from therapy (Brandi et al., 2006). This can be due to the production of toxic metabolites resulting from bacterial metabolism of the administered compounds. As discussed in the previous chapter, removal of GlcA from SN38-G leads to reactivation of SN38 resulting in adverse side effects to the patient (Wallace et al., 2015). Thus, inhibiting the production of GUS enzymes prevents intestinal toxicity and stabilizes the antitumor efficacy of irinotecan (Bhatt et al., 2020).

Taken together, these results suggest that the presence of some bacteria can lead to increased treatment-related side effects and toxicity. On the other hand, therapy efficacy can also be modulated by the gut microbiota (Taper and Roberfroid, 2005; Iida et al., 2013; Viaud et al., 2013). Interestingly, there seems to be a dual role of bacteria in cancer therapy as some studies report a synergistic effect of microbiota and drug efficacy, while others show the presence of bacteria to compromise therapy. More than 10 years ago, it was shown that supplementation of a diet rich in inulin or oligofructose led to growth inhibition of a transplantable tumour mouse model (Taper and Roberfroid, 2005). Inulin and oligofructose are fructans shown to promote the growth of *Bifidobacteria* in the gut (Gibson et al., 1995). The efficacy of 6 different chemotherapeutic drugs (5-FU, Doxorubicine, Vincristine, Cyclophosphamide, Methotrexate, Cytarabine) was potentiated by the addition of these supplements to the animals’ diet, suggesting a prebiotic effect of inulin and oligofructose (Taper and Roberfroid, 2005). In a preclinical model in which mice were injected with CRC cells, tumours showed resistance to gemcitabine treatment. The authors of this study reported that *Gammaproteobacteria* present in the tumour have the capacity to metabolize the drug into its inactive form, rendering the therapy ineffective (Geller et al., 2017). The therapeutic effect of gemcitabine was rescued when mice were treated with the antibiotic ciprofloxacin in combination with gemcitabine (Geller et al., 2017). In another study, by treating CRC cells with Oxaliplatin and 5-FU in combination with *F. nucleatum*, Yu and colleagues (2017) showed that bacteria-positive cells are resistant to the therapy compared to controls. To achieve this, *F. nucleatum* induces autophagy by stimulating the expression of pULK1, ULK1, and ATG7 proteins, rendering the therapy less effective in CRC cells (Yu et al., 2017). In addition, germ-free or antibiotic treated mice showed resistance to cyclophosphamide treatment, and have a poorer anti-tumour response in a sarcoma allograft mouse model in comparison to SPF mice (Viaud et al., 2013). In another study (Iida et al., 2013), mice transplanted subcutaneously with three different tumour cell lines (including a colon cancer cell line) harbouring a normal gut microbiota (WT) or absence of intestinal bacteria (antibiotic treated), were treated with different immunotherapy and chemotherapy regimens. Strikingly, antibiotic treatment impaired both therapies’ efficacy leading to higher tumour volumes and decreased survival of the animals when no microbiota is present (Iida et al., 2013). This response was shown to be dependent on TNF production by tumour-associated myeloid cells followed by a CD8^+^ T cell response. By correlating faecal microbiota composition with TNF production in antibiotic treated mice, the authors identified *A. shahii* species from the genus *Alistipes* to bear the strongest association. Thus, oral administration of *A. shahii* to microbiota-depleted mice reconstituted the ability of tumour-associated myeloid cells to produce TNF leading to an anti-tumour response (Iida et al., 2013). In line with this, a recent study identified two SCFAs (pentanoate and butyrate) as enhancers of adoptive cell therapy in cancer (Luu et al., 2021). By treating mice injected with B16OVA melanoma cells, with CD8^+^ T cells previously exposed to butyrate or pentanoate, the authors reported decreased tumour volume and weight compared to non-treated T cells. The mechanism through which SCFAs achieve this effect in T cells is by inhibition of class I histone deacetylases (HDACs) and upregulation of mTOR complex, a key regulator of cell growth and metabolism of immune cells (Luu et al., 2021). In order to test this approach in different treatment settings, the authors used CD8^+^ CAR T cells that recognize ROR1, a molecule highly expressed in epithelial tumours, to treat mice transplanted with ROR1-expressing pancreatic tumour cells. Treatment of the tumour bearing mice with ROR1-CAR T cells previously exposed to pentanoate led to tumour regression in these animals (Luu et al., 2021).

Faecal Microbiota Transplantation (FMT) was first used in 1958 to treat *Clostridium difficile* infection (CDI) (Eiseman et al., 1958). By helping restoring a beneficial microbiome in infected patients, it was possible to treat up to 80% of all CDI cases. FMT led to effective results in several other conditions such as IBD, diabetes and even autism and therefore became a promising treatment option (Gupta et al., 2016). Given its safety, the advantages of this strategy were also addressed as a way to ameliorate adverse effects from radiotherapy treatment. Radiotherapy is used as first line treatment option in combination with chemotherapy (chemoradiotherapy) for CRC treatment (Dekker et al., 2019). As a result of this treatment, patients may experience various side effects, including bone marrow and gastrointestinal toxicity. Preclinical studies showed that bacteria could ameliorate the side effects of radiotherapy treatment regimens (Cui et al., 2017). FMT from young healthy mice to irradiated mice with ionizing radiation greatly improved the survival of the latter in comparison to non-FMT irradiated controls. Strikingly, the best therapeutic outcomes were achieved when sex-matched donors were used for the FMT strategy (Cui et al., 2017). Moreover, administration of specific bacteria such as *Lactobacillus rhamnosus* to mice undergoing radiotherapy was shown to elicit a protective effect in the intestinal mucosa of test subjects (Ciorba et al., 2012). Clinical studies enrolling cancer patients under radiotherapy have shown that the use of probiotics led to a decrease of radiation-induced gut toxicity, such as diarrhea (Ma et al., 2019). Moreover, the gut microbiome was also recently shown to impact radiotherapy efficacy in distinct preclinical cancer mouse models (Shiao et al., 2021). Interestingly, radiotherapy treatment of antibiotic-treated mice (bacteria specific) failed to delay tumour growth in 2 orthotopic mouse models of breast and melanoma cancer, respectively, in comparison to mice harbouring a normal microbiota (Figure 1I). Conversely, ablation of fungal communities in the gut improved survival of animals and elicited a strong anti-tumour response after radiation treatment in the aforementioned cancer models. The authors further concluded that the treatment with antifungal antibiotics prior to treatment with ionizing radiation leads to a reduction of CD206^+^F4/80^+^ suppressive macrophages and a sharp increase of Granzyme B expressing CD8^+^ T cells. These molecular settings further enhance the immune response against the tumour. Using the same cancer models, this study elegantly highlights the opposing roles of two very distinct organism types (bacteria and fungi) inhabiting the gut in relation to the effectiveness of radiotherapy (Shiao et al., 2021) (Figure 1I).

The future of cancer therapy is bound to explore the dual role of gut bacteria in drug responses: on one hand, bacteria, as a result of their metabolism can aggravate the side effects of therapy. On the other hand, the presence of bacteria is crucial for the success of cancer therapy. Recent studies in which the effects of fungi in disease are also addressed (Aykut et al., 2019; Limon et al., 2019; Iliev and Cadwell, 2021; Shiao et al., 2021) further widens the complexity of the relationship between external commensal organisms with host immune cells. These findings raise the possibility that probiotics and other microbiota-modulating compounds could be used as adjuvant therapy for cancer treatment.

## Bacteria in Cancer Immune Checkpoint Blockade Therapy

The concept of immunotherapy relies on the recognition of tumour cells expressing a specific antigen of the major histocompatibility complex (MHC) by T cells, through its receptors (TCRs) (Waldman et al., 2020). Several molecules are known to regulate this complex biological process. Among them, the cytotoxic T lymphocyte-associated protein 4 (CTLA-4), programmed cell death 1 (PD-1), and PD-1 ligand (PD-L1) received great attention in recent years due to their use in cancer therapy. The function of these molecules is complementary and they act in order to ensure that T cell responses preserve self-tolerance and protect the body from pathogens and neoplasia development (Waldman et al., 2020). Using the immune system to elicit a therapeutic effect against tumour cells is the basis of the concept of immune checkpoint blockade (ICB) therapy. Targeting CTLA-4 (ipilimumab), PD-1 (nivolumab) or its ligand PD-L1 (pembrolizumab) has been shown to be extremely effective in the treatment of a variety of advanced cancers including melanoma and non-small cell lung cancer (Hodi et al., 2010; Brahmer et al., 2012). Many other cancer types however show resistance to this type of compounds thereby compromising the overall therapy efficacy. Given the great potential of ICB therapy in cancer, the identification of the reason (or reasons) leading to the acquisition of resistance upon treatment is of the utmost importance in the field. Recent studies have suggested that the composition of the gut microbiota can predict the effectiveness of ICB therapy in both human patients and animal models (Sivan et al., 2015; Vétizou et al., 2015; Gopalakrishnan et al., 2018; Matson et al., 2018; Routy et al., 2018; Pinato et al., 2019; Tanoue et al., 2019). In fact, antibiotic treatment of patients with a wide variety of solid tumours (including non–small cell lung cancer and melanoma), prior to ICB therapy is associated with a worse treatment response and overall survival (OS) (Pinato et al., 2019). Consistently, dysbiosis was shown to lead to acquired resistance to ICB therapy (Routy et al., 2018). Moreover, the therapeutic efficacy of ipilimumab, an antibody directed towards CTLA-4, was shown to be influenced by the microbiota composition of the host both in humans and in several preclinical tumour models (Vétizou et al., 2015). In the latter, the authors also reported that germ-free and antibiotic-treated mice did not show response to CTLA-4 blockade therapy. Interestingly, inoculation of specific bacterial species in these mice (*B. fragilis* and/or *B. thetaiotaomicron* and *Burkholderiales*) rescued the therapeutic effect of ipilimumab in a T1H dependant manner (Vétizou et al., 2015). In another seminal study conducted by Sivan et al. (2015) the authors further uncovered the ways through which the microbiome can modulate therapy response in solid tumours (Sivan et al., 2015). When cohousing animals with similar tumour models from different animal facilities, the authors noted that the previously observed differences (before co-housing of the animals) in terms of tumour growth, were then eliminated. FMT experiments from one group of animals to the other showed similar results highlighting the importance of microbiota in tumorigenesis. Additionally, treatment of these animals with antibodies targeting PD-L1 resulted in slower tumour growth and this response was found to be mediated by the increased induction and infiltration of CD8^+^ T cells. *Bifidobacterium* was identified to be the responsible bacterial community for this effect, that, in combination with anti-PD-L1 therapy, almost abolished tumour growth (Sivan et al., 2015). The possibility of the existence of a protecting and also therapy-prone microbiota is again elegantly supported by the work of Routy et al. (2018) in which the authors used a similar approach to dissect the influence of the microbiota in the efficacy of ICB therapy (Routy et al., 2018). FMT from cancer patients who responded to ICB therapy into germ-free or antibiotic-treated mice, greatly increased the efficacy of PD-1 blockade in different cancer models. When analysing the microbiota of cancer patients who responded to ICB, or did not, one commensal bacteria (*A. muciniphila*) was found to be enriched in responders compared to non-responders and its presence associated with longer patient progression free survival (PFS). Moreover, oral supplementation of mice that were subjected to FMT from patients that did not respond to ICB with *A. muciniphila*, restored the responsiveness of PD-L1 blockade therapy (Routy et al., 2018). In accordance to these findings, treating mice with metformin, a drug primarily used to treat type-2 diabetes which suppresses glucose production in the liver, was found to improve the abundance of *A. muciniphila* in the gut of aged obese mice. Inoculation of obese mice with *A. muciniphila* led to improved body weight and lipid profiles in these animals (Lee et al., 2018). Combined, these findings underlie possible synergistic effect between therapeutic compounds and bacteria in disease treatment. In another study (Matson et al., 2018), using a similar methodology, the authors identified *Bifidobacterium longum*, *Collinsella aerofaciens*, and *Enterococcus faecium* to be more abundant in the stool of melanoma patients that responded to anti PD-L1 therapy compared to non-responders. FMT from responders into germ-free mice induced a T cell-dependant response against tumour cells in an orthotopic melanoma model, further enhancing the efficacy of ICB (Matson et al., 2018). Taken together, these results demonstrate the profound effect of the gut microbiota on the efficacy of cancer immunotherapy *in vivo*.

Several studies suggested a positive therapeutic effect of ketogenic diet (KD) in many diseases including cancer (Branco et al., 2016). KD is characterized by high-fat, moderate-protein content while minimizing the intake of carbohydrates, ultimately leading to an increase of ketone bodies (KB) production. By reducing glucose availability and providing KB as an alternative energy source, it would be possible in theory to counteract the Warburg effect in cancer cells, characterized by a bioenergetic shift from oxidative phosphorylation towards glycolysis (Warburg et al., 1927). Mice kept in a KD showed changes in the microbiota composition (Ferrere et al., 2021). More specifically there was an overrepresentation of the bacteria *Akkermansia muciniphila*, *Ruthenibacterium lactatiformans*, and *Pseudoflavonifractor capillosus.* Strikingly, KD attenuated tumour growth in an orthotopic melanoma mouse model compared to mice fed a normal diet. By combining KD with ICB therapy (anti–PD-1 or anti–CTLA-4) the anti-tumour effects of the treatment were potentiated even in tumours that showed previous resistance to ICB drugs. In combination with ICB, KD regimens induced the upregulation of PD-1 and CTLA-4 on CD8^+^ T cells, and at the same time prevented the expression of their ligands on splenic macrophages. This in turn leads to a prolonged systemic T cell activation and thus to an increased immune response against the tumour (Ferrere et al., 2021). In summary, these results show how diet can influence the microbiota and how these effects impact on the predisposition of cancer development. Manipulating the microbiota through diet allows concomitant modulation of cancer therapy efficacy, setting the stage for a new perspective on possible cancer treatment options.

The benefits of combined immune checkpoint blockade (CICB) therapy targeting CTLA-4 and PD-1, for the treatment of melanoma has proved highly effective in a subset of patients (Larkin et al., 2015). Nonetheless, a significant proportion of subjects experience immune-related adverse events as a consequence of this treatment (Sznol et al., 2017). A recent study suggests that CICB toxicity may be mediated bacteria that lead to increased production of IL-1β in this cancer model (Andrews et al., 2021). IL-1β is produced by monocytes in response to commensal microbiota and induces inflammation in the intestine (Seo et al., 2015). By analysing the microbiota of 77 melanoma patients that developed immune-related events, the authors identified a higher abundance of *Bacteroides intestinalis* in the faeces of these patients compared to patients that did not show adverse effects. Concomitantly, oral gavage of this bacteria into antibiotic-treated mice was strongly associated with ileal *Il1b* transcription. Interestingly, CICB treatment of these mice led to a stronger overrepresentation of *Bacteroides intestinalis* over other *Bacteroides* species confirming the causal relationship between treatment and bacteria. As a final proof, the authors performed FMT from healthy human donors harbouring high levels of endogenous *Bacteroides intestinalis* into tumour-bearing mice*.* This led to induction of *Il1b* expression in the intestine following CICB treatment of a melanoma mouse model (Andrews et al., 2021).

Only a small percentage of CRC patients respond to ICB therapy. Surprisingly, combination of conventional chemotherapeutic agents with ICB have not so far proven superior to chemotherapy alone for the treatment of metastatic disease (Zou et al., 2016). Recent seminal studies using preclinical animal models suggested that ICB therapy efficiency in CRC can be influenced by the gut microbiota (Tanoue et al., 2019; Roberti et al., 2020; Mager et al., 2020). Of note, a consortium of 11 bacterial strains derived from healthy human donors were shown to increase the frequency of colonic IFNγ^+^ CD8^+^ T cells upon inoculation into germ-free mice (Tanoue et al., 2019). IFNγ^+^ CD8^+^ T cells contain subsets that expressed tissue-resident memory T cell marker CD103, and granzyme B (GrB), a key effector molecule of cytotoxic T cells, and are therefore capable of inducing an anti-tumour response (Figure 1J). Furthermore, the effect of anti-PD-1 therapy in mice engrafted with MC38 colon adenocarcinoma cells, was markedly ameliorated upon colonization with these 11 bacteria species (Tanoue et al., 2019). Using a similar CRC xenograft mouse model, Roberti at al. (2020) were successful in delaying tumour growth in mice treated with Oxaliplatin plus nivolumab in combination with either the *B. fragilis* or *E. ramosum* bacteria species. These bacteria strongly induced the production of IL-1β and IL-12p70 by DCs in the tumour microenvironment. This in turn drove the differentiation of T cells into Th1 cells, eliciting a response against the tumour (Roberti et al., 2020). In line with these results, another study (Mager et al., 2020) identified further bacterial species involved in the modulation of the efficacy of ICB treatment in CRC. The analysis of the microbiome of mouse AOM/DSS-induced colorectal tumours that responded to ICB, revealed *Bifidobacterium pseudolongum* to be differentially abundant in responders compared to non-responders. The authors then colonized tumour-bearing germ-free mice (injected with MC38 CRC cells) with this bacteria strain and treated them with ICB antibodies. Combinatorial treatment of bacteria and anti-PDL1, or anti-CTLA4, elicited an anti-tumour response shown by reduced tumour size and increased T cell activation in these animals (Figure 1K). Surprisingly, replacement of *Bifidobacterium pseudolongum* by inosine, a metabolite produced by this bacterium, in combination with anti-CTLA4 and CpG (a common immunostimulatory anti-cancer compound) led to similar results in this cancer model (Mager et al., 2020) (Figure 1K).

Clinically, microsatellite instable (MSI-H) tumours show better response rates to ICB when compared to microsatellite stable (MSS) tumours (Le et al., 2017; Overman et al., 2017). The MSI phenotype is caused by DNA mismatch repair (MMR) deficiencies that trigger the generation of frameshift mutations in several loci of the genome. These mutations can give rise to neoantigens in tumour cells that stimulate immune responses in patients (Kloor and von Knebel Doeberitz, 2016). In line with this, MSI-H tumours present immune-related features, including more immune cell infiltration, upregulation of immune-related genes and higher immunogenicity. It is, therefore, plausible to assume that, given the established relationship between the gut microbiota and immune cells, certain bacteria are likely to interfere in this process by modulating the efficacy of ICB. More studies including the use of MSI-H preclinical cancer models could help explain the low efficacy rate of ICB in MSS tumours and also why only 30–50% of MSI-H tumours show improved therapy responses.

Two recent studies took a leap forward in addressing one of the biggest problems of ICB treatment: acquired therapy resistance (Baruch et al., 2021; Davar et al., 2021). The clinical trial performed by Baruch et al. included previously diagnosed patients with metastatic melanoma who had progressed on at least one line of anti-PD-1 therapy (Baruch et al., 2021). These patients were subsequentially treated with FMT from two donors who had been treated with anti-PD-1 monotherapy for metastatic melanoma, and achieved a complete response for at least 1 year, together with re-induction of anti-PD-1 immunotherapy. Strikingly, 3 out of the 10 patients treated with this combinatorial therapy regime showed clinical responses including two partial and one complete response (Baruch et al., 2021). In another clinical trial with a similar study design, Davar et al. (2021) also showed that anti-PD-1 therapy acquired resistance could be overcome in 40% of patients by FMT treatment from individuals who had previously benefited from the same ICB therapy (Davar et al., 2021). Furthermore, responders showed a boosted immune response, reflected by increased CD8^+^ T cell activation, and decreased frequency of interleukin-8-expressing myeloid cells (Davar et al., 2021).

It becomes evident that more and more bacteria species will be implicated as modulators of cancer therapy in the years to come, and with that, an improvement in how the best therapeutic outcomes can be achieved. It should be clear, however, that other variables such as diet, geographical location, lifestyle behaviours (such as tobacco smoking) might affect the microbiota composition and its metabolic profile. The concept of a “personalized microbiota” is currently being explored by pharma and biotech companies and several clinical trials using bacteria are ongoing including several cancer models (Zipkin, 2021).

## Using Bacteria to Fight Cancer

Due to their inherent biology, microbes are good at synthesizing active molecules including many therapeutic compounds. In fact, this is a feature currently explored by biotech companies, which commercialize a plethora of biological compounds such as antibiotics, vitamins or antigens produced by genetically engineered microbes (Pedrolli et al., 2019). The basis of this approach resides on the fact that microbes can be used as shuttles or “chassis” to which one can load with specific cargo to elicit a desirable biological effect (Charbonneau et al., 2020). In theory, a suitable chassis such as *E. coli* or *L. lactis* show a high prevalence in the normal human gut but should be non-colonizing, and therefore, cleared shortly after administration (Human Microbiome Project, 2012; Pedrolli et al., 2019).

The use of the microbiota as direct therapy to tackle cancer and other diseases has also been assessed as a possibility and has shown promising results in recent years (Nelson et al., 2021; Braat et al., 2006). With the advent of genetic engineering technologies, it is now possible to use bacteria as a delivery system to selectively release therapeutic compounds into the tumour *in vivo* (Zhu et al., 2019; Riglar and Silver, 2018; Zhou et al., 2018; An et al., 2019; Chung et al., 2021; Chowdhury et al., 2019; Frahm et al., 2015). Since the first study employing this methodology in cancer (Frahm et al., 2015), several other groups turned their attention to this approach. Of note, oral administration of P8 protein-producing *Pediococcus pentosaceus*, was shown to elicit an anti-tumour effect in two CRC mouse models, to a similar extent as with conventional chemotherapy (Chung et al., 2021) (Figure 1J). Furthermore, P8 treatment was shown to also alleviate the change from eubiosis to dysbiosis induced by AOM/DSS in one of the models tested (Chung et al., 2021). In another elegant study, Chowdhury et al. (2019) achieved increased activation of tumour-infiltrating T cells *in vivo* using different tumour models by transforming a non-pathogenic *E. coli* strain with a targeting molecule against CD47 (Chowdhury et al., 2019). As a direct consequence, tumour regression was achieved in all cancer models. CD47 is an anti-phagocytic receptor overexpressed in several human cancers and its blockade not only increases phagocytosis of cancer cells *in vitro* but also promotes activation of T cells against tumours *in vivo* (Chowdhury et al., 2019; Sockolosky et al., 2016). By transforming bacteria with a plasmid that encodes a synchronized lysis circuit (SLC) molecule plus a nanobody antagonist of CD47 (CD47nb), the authors were able to selectively release the therapeutic agent in to tumour cells *in vivo*. This approach avoids common comorbidities associated with systemic CD47 blockade reported in human trials (Chowdhury et al., 2019; Advani et al., 2018). Another seminal example of how one can use bacteria to selectively target CRC cells in an organism comes from the study of Ho et al. (2018). Briefly, by reprogramming commensal *E. coli*, to selectively bind the heparan sulfate proteoglycan (HSPG) located on the surface of cancer cells, the authors could, once again, successfully direct bacteria to the malignant lesion. Once there, bacteria were edited to secrete myrosinase, an enzyme that mediates the conversion of dietary glucosinolate to sulforaphane, and was shown to inhibit growth and promote apoptosis of cancer cells. As a result, tumour clearance was achieved in an AOM/DSS-induced cancer mouse model (Ho et al., 2018) (Figure 1J). Interestingly, mice treated with this bacterial system in combination with a cruciferous diet showed the best outcome regarding tumour prevention (Ho et al., 2018), which highlights, once more, the role of diet as a modulator of cancer treatment and prevention.

In line with this, perhaps in a more conventional way, bacteria can also be used as probiotics for therapeutic purposes. Probiotics by definition are microorganisms that confer health benefits when administered in controlled amounts (Geier et al., 2006). Even though the use of probiotics is not sufficient to cure or eradicate disease, its use has shown very convincing results as an adjuvant therapeutic approach in order to ameliorate side effects from both maladies and therapy. In fact, its use was reported to aid in several pathologic states including bacterial infection, by diminishing the colonization of pathogenic bacteria (Geier et al., 2006) and inflammation, by supressing inflammatory pathways or switching the phenotype of macrophages from M1 (pro inflammatory) to M2 (immunosuppressive) (Sichetti et al., 2018). The immunomodulatory properties of probiotics are further highlighted in the work of Chen et al. (2012). The inoculation of the bacterial strain *Lactobacillus acidophilus NCFM* was shown to downregulate MHC class I molecules in tumour cells of CT-26-implanted mouse models, resulting in an increased antitumour T-cell response (Chen et al., 2012). Moreover, certain bacterial species such as *Lactobacillus rhamnosus, Lactobacillus plantarum* and *Escherichia coli* can improve gut barrier function which is often disrupted in CRC (Kumar et al., 2017). The use of probiotics is generally accepted as a safe procedure, nonetheless attention must be placed in subjects with underlying medical conditions for instance immunosuppressed patients. In this setting the translocation of viable bacteria to a donor can lead to infections due to the poor capacity of the immune system to eradicate pathogenic bacteria from the host (Doron and Snydman, 2015). The prolonged use of probiotics could theoretically lead to horizontal gene transfer events where mobile genetic elements are disseminated within bacterial communities and render bacteria resistant to antibiotics (Jacobsen et al., 2007).

Bacteriophages are a class of prokaryotic viruses with the ability to infect host bacterial cells. Once inside the bacteria, bacteriophages replicate and produce endolysins that destroy the bacterial cell wall allowing the release of their viral particles (lytic phages). Lysogenic phages on the other hand, have the ability to integrate the bacterial genome and propagate their genetic information to the next generations. In recent years bacteriophages, given their versatility of use and specificity in infecting a plethora of cell types, have been explored as drug delivery systems for cancer treatment (Yacoby and Benhar, 2008). By employing a biopanning strategy it is possible to scan both *in vivo* and *in vitro* systems for the identification of cell surface-interacting peptides and uncover novel tumour-associated antigens for the design of targeted delivery systems. Several studies showed the feasibility of this strategy, reporting promising treatment approaches in different cancer models, either by conjugation of bacteriophages with therapeutic compounds or nucleic acids (Shadidi and Sioud, 2003; Cai et al., 2008; Du et al., 2010) (Figure 1L). By packaging a siRNA against focal adhesion kinase (*FAK*) gene in an EGF-targeting bacteriophage, Cai et al. were able to inhibit cell growth and invasiveness in an EGFR-overexpressing lung carcinoma cell line (Cai et al., 2008). Moreover, after injecting a phage library into a hepatocarcinoma xenograft mouse model, Du and colleagues (Du et al., 2010) identified a phage clone that in conjugation with Doxorubicin elicited a strong anti-tumour activity in the same model. Bacteriophages can also be used in combination with other delivery systems, for instance adeno-associated viruses (AAV) to further increase target specificity and infection efficiency in a safe manner (Hajitou et al., 2006; Hajitou et al., 2008). Only a few examples of the use of bacteriophages as therapeutic tools in cancer are highlighted in this review, since this topic has been extensively reviewed elsewhere (Foglizzo and Marchiò, 2021).

Current advances in understanding the evolution of a tumour as well as its treatment implications have improved the clinical outcome of cancer patients in recent years. Increasing evidence suggests that bacteria play an important role in determining the effectiveness of therapy. However, there are still concerns about the side effects and drug resistance associated with current treatment programs. Although some side effects are observed in early clinical trials when using live bacteria as therapeutic agents, they still show far less toxicity in comparison to conventional chemotherapy regimens in different cancer types (Toso et al., 2002; Maciag et al., 2009).

## Concluding Remarks

CRC is regarded as a genetic disease, as mutations are the events responsible for the transformation of a normal cell into a cancer cell. Cancer progression further requires a plethora of cell-intrinsic and cell-extrinsic processes that ultimately disguises the tumour from immune regulation, allowing at the same time its proliferation and ultimately invasion to distant organs (Hanahan and Weinberg, 2011). The studies included in this review link bacteria to dysregulation of known cancer pathways, thereby complementing the current knowledge about cancer initiation and progression. Furthermore, they show the importance of the genetic and metabolic features of bacteria, and how these affect human host cells in CRC. Mounting evidence further implies that the gut microbiota determines the effectiveness of conventional and targeted therapy drugs but can also be responsible for its adverse side effects. Thus, we suggest the identification of microbiota-specific features in a given clinical setting to be of the utmost importance for comprehensive disease assessment. Moreover, since these features could potentially be used as biomarkers of disease prognosis and therapy response outcomes, we propose the concept of personalised medicine to be revisited. Clinical management of patients should in the near future include data from both the patient and their associated microbiota when evaluating treatment decisions. We estimate that the use of clinical studies with proper standardisation and grouping according to the genetic and metabolic aspects of microbiota will help explain the discrepancies in therapy efficacies of different patients with similar molecular and histological cancer subtypes.
